# A Model of Multi-Finger Coordination in Keystroke Movement

**DOI:** 10.3390/s24041221

**Published:** 2024-02-14

**Authors:** Jialuo Lin, Baihui Ding, Zilong Song, Zheng Li, Shengchao Li

**Affiliations:** Key Laboratory of Mechanism Theory and Equipment Design, Ministry of Education, Tianjin University, Tianjin 300350, China; linjialuo@tju.edu.cn (J.L.); songzilong@tju.edu.cn (Z.S.); 2021201252@tju.edu.cn (Z.L.); shengchao_li@tju.edu.cn (S.L.)

**Keywords:** multi-finger coordinated keystroke, Leap Motion sensors, coordination, BP neural network, sparrow search algorithm, genetic algorithm

## Abstract

In multi-finger coordinated keystroke actions by professional pianists, movements are precisely regulated by multiple motor neural centers, exhibiting a certain degree of coordination in finger motions. This coordination enhances the flexibility and efficiency of professional pianists’ keystrokes. Research on the coordination of keystrokes in professional pianists is of great significance for guiding the movements of piano beginners and the motion planning of exoskeleton robots, among other fields. Currently, research on the coordination of multi-finger piano keystroke actions is still in its infancy. Scholars primarily focus on phenomenological analysis and theoretical description, which lack accurate and practical modeling methods. Considering that the tendon of the ring finger is closely connected to adjacent fingers, resulting in limited flexibility in its movement, this study concentrates on coordinated keystrokes involving the middle and ring fingers. A motion measurement platform is constructed, and Leap Motion is used to collect data from 12 professional pianists. A universal model applicable to multiple individuals for multi-finger coordination in keystroke actions based on the backpropagation (BP) neural network is proposed, which is optimized using a genetic algorithm (GA) and a sparrow search algorithm (SSA). The angular rotation of the ring finger’s MCP joint is selected as the model output, while the individual difference information and the angular data of the middle finger’s MCP joint serve as inputs. The individual difference information used in this study includes ring finger length, middle finger length, and years of piano training. The results indicate that the proposed SSA-BP neural network-based model demonstrates superior predictive accuracy, with a root mean square error of 4.8328°. Based on this model, the keystroke motion of the ring finger’s MCP joint can be accurately predicted from the middle finger’s keystroke motion information, offering an evaluative method and scientific guidance for the training of multi-finger coordinated keystrokes in piano learners.

## 1. Introduction

Piano playing involves a variety of complex and dexterous finger movements, imposing high demands on the performer’s ability to coordinate finger movements [1]. From an anatomical perspective, the flexion movements of human fingers are primarily controlled by the deep and superficial flexor muscles connected to the fingers via tendons [2]. However, due to the ring finger’s tendons being closely connected to adjacent fingers [3], it is significantly influenced by the enslaving effect [4], which affects the coordination and independence of the ring finger relatively [5]. Previous studies have commonly reported that the middle and ring fingers generate greater motor overflow compared to the index and little fingers in terms of movement independence, which means that when the middle or ring finger is in motion, the other fingers may also involuntarily produce movement [6]. Despite inherent neural and biomechanical limitations, professional pianists are still able to execute keystrokes with high precision at varying playing speeds [7]. Humans utilize the corticospinal system to control finger movements for mastering piano playing, wherein the nervous system activates the relevant muscles via neuromuscular junctions, enabling interaction between the skeletal system and the piano [8,9]. Compared to non-professionals, long-term trained professional pianists exhibit structural and functional changes in the cerebral cortex responsible for hand movements [10]. To elaborate on human piano keystroke behavior, a series of physiological experiments have been conducted, with extensive studies on the movements of professional pianists, particularly their motor coordination in rhythmic keystroke actions. Shinichi Furuya et al. conducted a comparative analysis of the kinematics and muscle activity of the hands of professional and amateur pianists during the alternate striking of two keys using the thumb and little finger. The research unveiled discernible disparities in coordination strategies between professional and amateur musicians, with professional pianists demonstrating reduced flexion velocity in both the thumb and little finger [11]. Shinichi Furuya and John F. Soechting observed that the finger movement coordination patterns at different tempos exhibited similarity in terms of the kinematic parameters of finger motion and the sequence of musical notes. This observation suggests that, in piano performance at varying tempos, the coordination of finger movements remains consistent in certain aspects [12]. Wang et al.’s research introduces a computational model to investigate coordinated upper-limb movements in piano playing, emphasizing the role of central pattern generators and musculoskeletal dynamics in facilitating expressive and efficient performance [13]. However, most of these studies merely observe and analyze the coordination phenomena, offering little guidance for practical engineering applications. Additionally, deterministic motion coordination relationships are needed in areas such as limb coordination, muscle energy consumption monitoring, and robotic adaptive control [14,15,16]. Given the interconnected tendons of the ring finger and adjacent fingers, with the middle and ring fingers exhibiting greater motion overflow characteristics, this study focuses on their coordinated keystroke actions in professional pianists, capturing these multi-finger coordinated movements and establishing a mathematical model.

Currently, the measurement of human hand movements can be categorized into contact methods and non-contact methods. Contact-based measurements often employ exoskeletons or data gloves to collect information on hand joint movements, offering direct and reliable data [17,18]. However, these methods necessitate individuals to wear external devices and involve adjustments based on variations in hand sizes. This can make the measurement process cumbersome and potentially impact the natural piano keystroke actions, leading to deviations in measurement data from actual movements. Non-contact measurements utilize multi-camera systems or vision-based 3D sensing devices (such as Kinect and Leap Motion) to recognize hand movements [19,20], eliminating the need for individuals to wear external devices and allowing for more natural movements. However, these methods may encounter occlusion issues during measurement, necessitating the construction of a dedicated motion measurement platform. Compared to multi-camera systems and Kinect, Leap Motion is a 3D motion-sensing device specifically developed for capturing human hand movements, capable of directly recognizing joint position information with a precision of up to 0.01 mm. Leap Motion is extensively used in fields including gesture recognition and motion capture [21,22].

In recent years, prediction methods based on artificial neural networks (ANNs) have achieved significant accomplishments in various research fields [23,24]. Benefiting from the powerful learning capacity and complex mechanisms for acquiring information relationships of ANNs, prediction methods based on ANNs possess stronger generalization abilities [25]. Among the various neural networks, backpropagation neural networks (BPNNs) are particularly noteworthy [26]. It is estimated that approximately 80% to 90% of neural network models utilize BPNNs or their derivative variants [27]. The BPNN, a multi-layer feedforward network model based on the error backpropagation algorithm, can approximate any complex nonlinear function to an infinite degree [28] and is considered one of the most accurate neural network models to date [29]. However, due to the random initialization of initial weights and thresholds, BP neural networks are prone to becoming trapped in local minima [30]. Some researchers have considered optimizing BPNN models using genetic algorithms (GAs) and sparrow search algorithms (SSAs), which have been successfully applied in nonlinear prediction domains such as transportation, industrial manufacturing, and cybersecurity [31,32,33].

Consequently, considering the impact of contact-based measurement devices on the natural piano keystroke actions of the hand, this study employs the Leap Motion sensor for non-contact measurement of the coordinated keystroke actions of the middle and ring fingers first. A personalized multi-finger coordinated keystroke action model was established based on the BP neural network, tailored to individual characteristics. Then, on the basis of this model, a cross-individual and cross-characteristic multi-finger coordinated keystroke action model was developed by incorporating individual participant features such as middle finger length, ring finger length, and years of piano training. Finally, the genetic algorithm and sparrow search algorithm were employed to optimize the model’s initial weights and thresholds, enhancing predictive accuracy and establishing a model applicable to various individuals.

## 2. Methods

### 2.1. Coordinate System for Hand Joints

**Figure 1:**
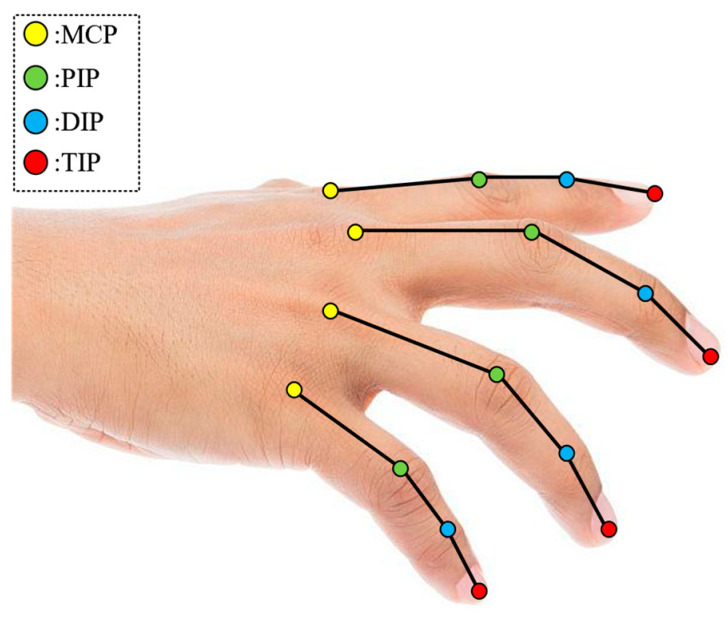
Physiological structure of fingers (except thumb).

As shown in Figure 1, the human fingers (excluding the thumb) consist of the metacarpophalangeal (MCP), proximal interphalangeal (PIP), and distal interphalangeal (DIP) joints [34]. The MCP joint has two degrees of freedom, while both the PIP and DIP joints each have one degree of freedom. During the execution of coordinated multi-finger keystroke actions, the coordinated movement of these finger joints ensures accuracy and agility in piano playing, which is vital for performance techniques. This coordination involves dynamic relationships between fingers, such as adjustments in angles and control of timing, all of which must be precisely matched to produce the correct keystroke actions.

**Figure 2:**
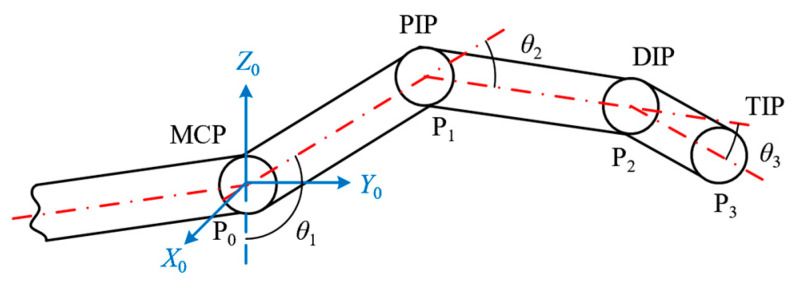
Simplified diagram of middle finger keystroke movements.

The action of multi-finger keystroke primarily involves flexion and extension movements, this paper only focuses on the flexion/extension degrees of freedom of the MCP joint. Taking the middle finger as an example, a motion model for the middle finger’s key-pressing action in multi-finger coordination was established, as shown in Figure 2. Herein, the central points of the MCP, PIP, DIP, and TIP joints are denoted as $P_{0}$, $P_{1}$, $P_{2}$, and $P_{3}$, respectively. $\vec{P_{0}P_{1}}$, $\vec{P_{1}P_{2}}$, and $\vec{P_{2}P_{3}}$, respectively, represent the proximal, middle, and distal phalanges. Using the center point $P_{0}$ of the MCP joint of the middle finger as the coordinate origin, a fixed coordinate system, denoted as {0}, was established, wherein the $X_{0}$ axis extends outward along the axis of rotation, the $Y_{0}$ axis horizontally to the right, and the $Z_{0}$ axis vertically upward. The middle finger is positioned within the $Y_{0}P_{0}Z_{0}$ plane. Within this plane, the angle $\theta_{1}$ (counterclockwise positive) between $\vec{Z_{0}P_{0}}$ and $\vec{P_{0}P_{1}}$ indicates the rotational angle of the metacarpophalangeal (MCP) joint, the angle $\theta_{2}$ (clockwise positive) between $\vec{P_{0}P_{1}}$ and $\vec{P_{1}P_{2}}$ represents the rotation angle of the proximal interphalangeal (PIP) joint, and the angle $\theta_{3}$ (clockwise positive) between $\vec{P_{1}P_{2}}$ and $\vec{P_{2}P_{3}}$ denotes the rotation angle of the distal interphalangeal (DIP) joint.

By calculating the hand feature point position data collected earlier, the corresponding joint rotation angle data can be obtained. Considering the unit vector of $\vec{Z_{0}P_{0}}$ as ${\vec{u}}_{Z_{0}P_{0}}$ and the known length of the proximal phalanx as $\left|\vec{P_{0}P_{1}}\right|$, the rotational angle ($\theta_{1}$) of the middle finger’s MCP joint position is given as Equation (1):

(1) $$\theta_{1}=\cos^{-1}({\vec{u}}_{Z_{0}P_{0}}\cdot \frac{\vec{P_{0}P_{1}}}{\left|\vec{P_{0}P_{1}}\right|})$$

where $\frac{\vec{P_{0}P_{1}}}{\left|\vec{P_{0}P_{1}}\right|}$ is the unit vector of $\vec{P_{0}P_{1}}$. ${\vec{u}}_{Z_{0}P_{0}}\cdot \frac{\vec{P_{0}P_{1}}}{\left|\vec{P_{0}P_{1}}\right|}$’s dot product is calculated by

(2) $${\vec{u}}_{Z_{0}P_{0}}\cdot \frac{\vec{P_{0}P_{1}}}{\left|\vec{P_{0}P_{1}}\right|}={\vec{u}}_{Z_{0}P_{0y}}\cdot \frac{{\vec{P_{0}P_{1}}}_{y}}{\left|\vec{P_{0}P_{1}}\right|}+{\vec{u}}_{Z_{0}P_{0z}}\cdot \frac{{\vec{P_{0}P_{1}}}_{z}}{\left|\vec{P_{0}P_{1}}\right|}$$

whereas ${\vec{u}}_{Z_{0}P_{0y}}$ and ${\vec{u}}_{Z_{0}P_{0z}}$ represent the y and z components of the unit vector ${\vec{u}}_{Z_{0}P_{0}}$, and ${\vec{P_{0}P_{1}}}_{y}$ and ${\vec{P_{0}P_{1}}}_{z}$ correspond to the y and z components of $\vec{P_{0}P_{1}}$. The angular velocity ($\omega_{1}$) and angular acceleration ($a_{1}$) data of the middle finger MCP joint are given as follows:

(3) $$\omega_{1}=\frac{d\theta_{1}}{dt}$$

(4) $$a_{1}=\frac{d\omega_{1}}{dt}$$

where t represents time. Following the calculation process for the MCP (metacarpophalangeal) rotation angle $\theta_{1}$ of the middle finger, a similar method can be applied to determine the MCP rotation angle $\beta_{1}$ of the ring finger.

### 2.2. Model Based on BPNN

**Figure 3:**
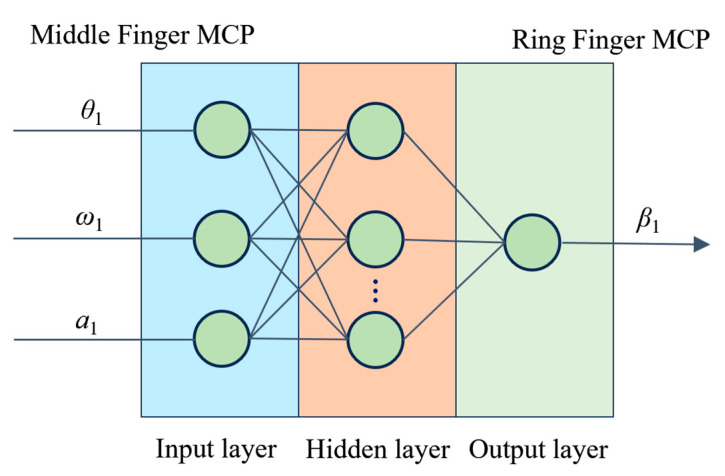
Individual multi-finger coordination keystroke neural network model.

Initially, this paper focuses on the establishment of a model for multi-finger coordinated keystroke actions in individuals. A three-layer BP neural network model is employed to model the coordination relationship of multi-finger coordinated keystroke actions in individuals, with the model input consisting of middle finger MCP joint motion information (MCP joint positional rotation angle, angular velocity, and angular acceleration data), and the ring finger MCP joint rotation angle as the model output, as illustrated in Figure 3. Drawing on previous research, the number of neurons in the hidden layer is determined to be 13 via a trial-and-error process [35]. During the model training process, the Levenberg–Marquardt algorithm [36] is utilized to ensure efficient optimization of model parameters and their convergence.

**Figure 4:**
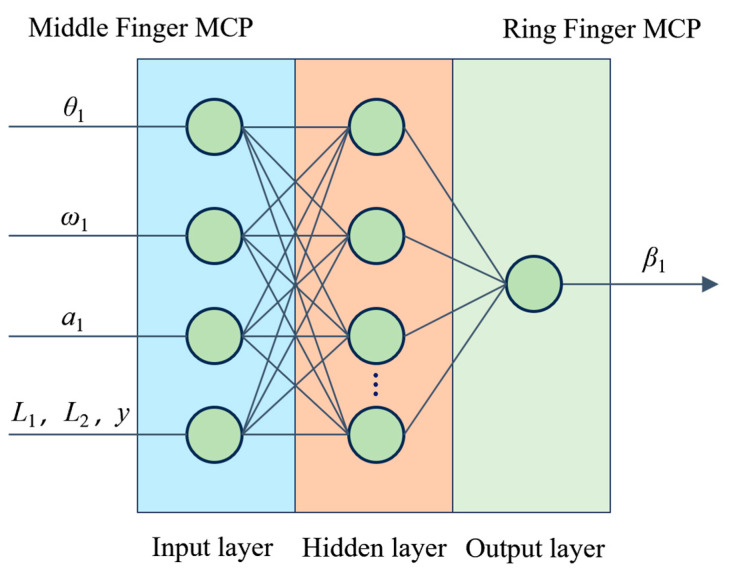
Multi-individual multi-finger coordination keystroke neural network model.

Due to individual characteristic differences (such as ring finger length, middle finger length, and years of piano training) [37], the coordination relationships in multi-finger piano keystroke actions [38], specifically referring to the precise coordination required among fingers to execute complex piano keystroke techniques, vary among individuals. Therefore, a multi-finger coordinated keystroke action model, which is built specifically for an individual participant, is only applicable to that participant. These models lack general applicability and are unsuitable for scenarios spanning multiple individuals. Addressing this issue, this paper introduces individual characteristic parameters like ring finger length, middle finger length, and piano training years, in addition to middle finger MCP joint motion information, to construct a general model applicable to various individuals. This model also employs a three-layer BP neural network, selecting middle finger MCP joint motion information (MCP joint positional rotation angle, angular velocity, and angular acceleration data) and individual variability information (ring finger length, middle finger length, and piano training years) as model inputs, with the ring finger MCP joint rotation angle as the output, as shown in Figure 4. The rationale for selecting these input variables is as follows: Firstly, the lengths of the middle and ring fingers reflect the anatomical characteristics of the hand, which have a direct influence on finger dexterity and coordination. In the execution of keystroke movement, finger length affects the range of motion, flexibility, and stability of the fingers. Secondly, the training duration of the participants reflects their experience and proficiency in specific skills. Prolonged training can lead to changes in neuromuscular coordination, affecting the accuracy and coordination of finger keystroke movements. Finally, data from the middle finger’s MCP joint provide direct information about the current state of finger movement. Due to the biomechanical and neurophysiological connections between the fingers, the keystroke movement status of the middle finger can directly impact adjacent fingers, such as the ring finger. Similar to the model previously established for individual participants, this neural network also employs a single hidden layer BPNN structure with 13 hidden neurons.

### 2.3. Optimization of the Model for Multi-Individual

**Figure 5:**
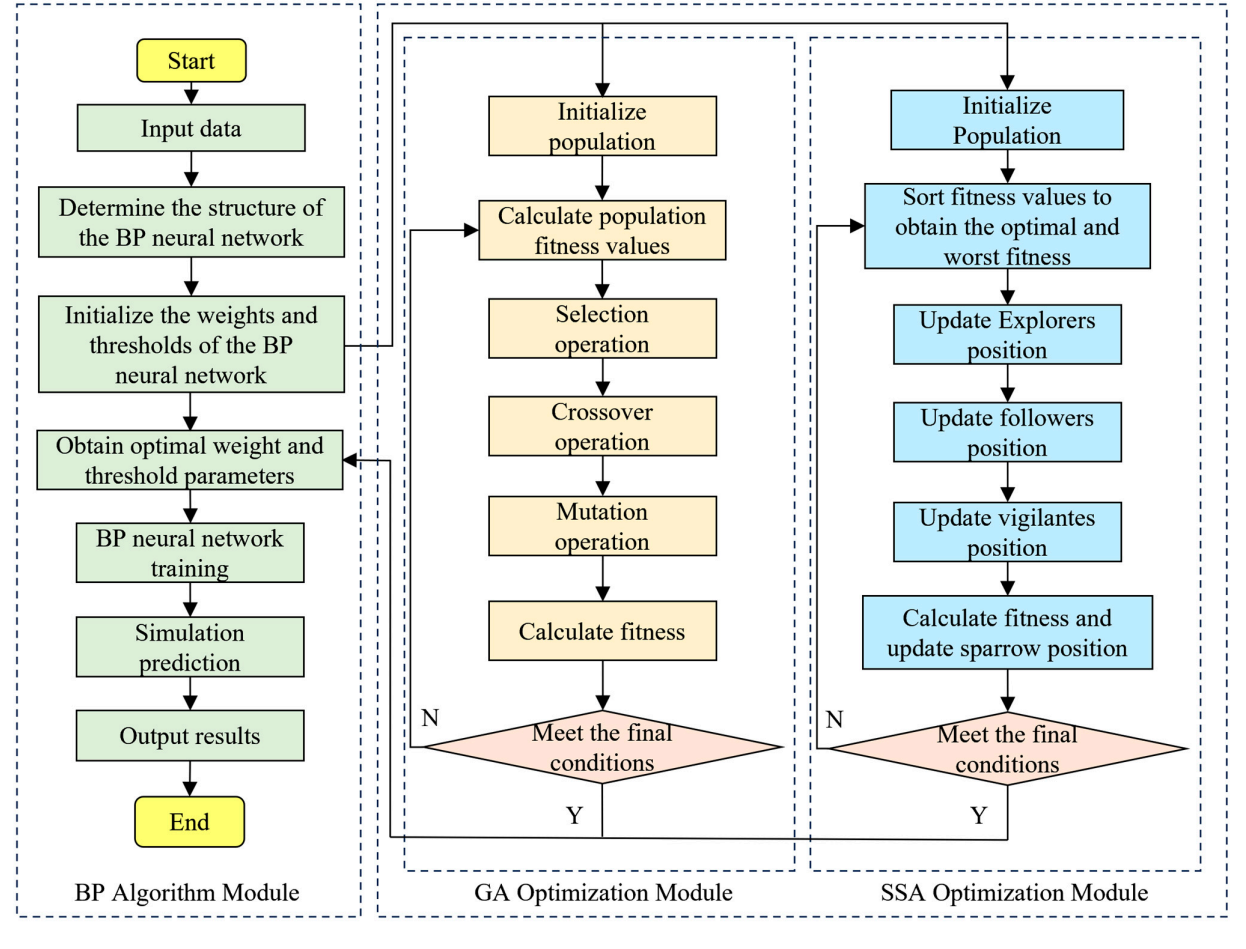
Flow chart of prediction for each model.

The genetic algorithm (GA) [39], a classic method for global optimization, draws its design inspiration from Darwin’s theory of evolution, employing mechanisms of natural selection and genetics, repeatedly executing operations like crossover, mutation, and selection to obtain optimal solutions. This algorithm demonstrates strong global search capability and robustness across various fields, particularly suitable for solving nonlinear, high-dimensional, and complex problems. The sparrow search algorithm (SSA), a novel optimization algorithm introduced by Xue et al. [40] in 2020, mimics the foraging and anti-predatory behaviors of sparrows in nature to search for optimal solutions. In the SSA, the sparrow population is divided into explorers, followers, and vigilantes, each role having a distinct function during the predation process. Explorers facilitate food finding for the entire sparrow population by identifying foraging areas and directions, followers acquire food by observing and following explorers, and vigilantes are responsible for alerting the group to danger, prompting strategic adjustments. Therefore, to address the issue of BP neural networks falling into local minima, this study utilizes both the GA and SSA to optimize the initial weights and thresholds of the BP neural network-based multi-individual, multi-finger coordinated keystroke models. Consequently, BP, GA-BP, and SSA-BP models have been developed, with their predictive processes illustrated in Figure 5.

Based on the analysis of the predictive processes of the BP, GA-BP, and SSA-BP models, the experiment employs a partially identical parameter setting to facilitate the comparison of the prediction accuracy of each model. All models employ a single hidden layer BPNN structure with 13 hidden neurons. The parameter settings for the GA-BP model are as shown in Table 1, with the fitness of individuals in the population represented by mean squared error. The parameter settings for the SSA-BP model, as indicated in Table 2, also utilize mean squared error as the fitness function.

## 3. Experiments and Results

### 3.1. Participant

Twelve professional pianists (seven males and five females, average age ± SD = 21.7 ± 4.3 years, and all right-handed), who had received several years of piano training, participated in this study. Each participant had a strong practice routine and had won awards in national or international piano competitions. The average lengths of their middle and ring fingers were 84.4 ± 4.3 mm and 79.1 ± 4.5 mm, respectively. The experimental procedures were clearly explained to the participants in accordance with the Helsinki Declaration, and written informed consent was obtained. The study was approved by the local ethics committee of Tianjin University.

### 3.2. Measurement Environment

**Figure 6:**
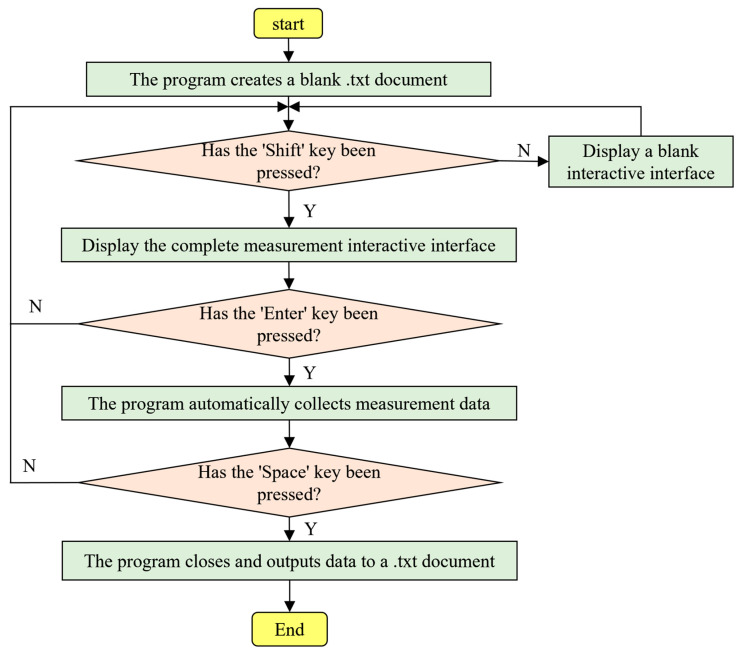
Program flow chart.

In order to facilitate the collection of data for multi-finger coordinated keystroke action, avoid obstructions during measurement, and ensure accuracy, a motion measurement platform was constructed, and the Leap Motion sensor underwent secondary development. A measurement program was written using Processing software in conjunction with the Java language to collect data on multi-finger coordinated keystroke action. Initially, the official device drivers and software development kit were downloaded and installed from the Leap Motion website, followed by the development of the measurement program using Processing software. This included the creation of a measurement interaction interface and the output and saving of measurement data, with the program flow illustrated in Figure 6.

**Figure 7:**
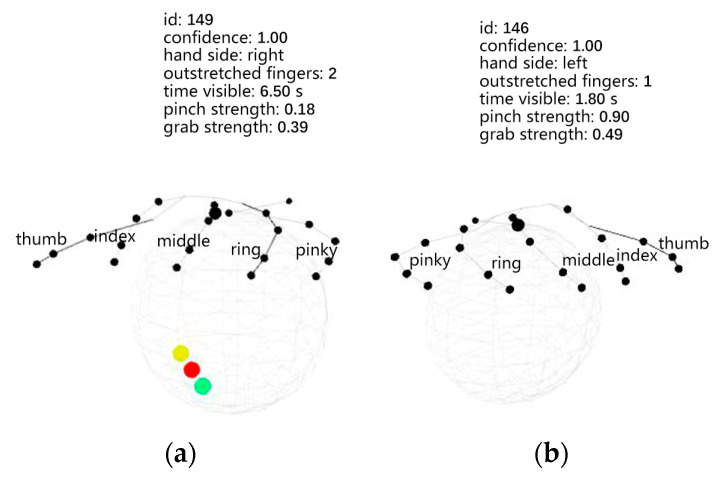
Measurement interfaces: (**a**) complete measurement interface; (**b**) incomplete measurement interface.

Upon initiation of the program, a blank .txt document is generated for storing the data obtained from the measurements. We can confirm whether the Leap Motion sensor has detected a complete human hand through the colored dots. When the Leap Motion sensor detects a complete human hand, it renders a hand model on the interface with three circular dots in yellow, red, and green, as shown in Figure 7a, indicating to the operator that data acquisition is proceeding normally. If the hand information captured is incomplete, the interface will lack the three circular dots, as depicted in Figure 7b, signaling to the operator that the data are inaccurate and the motion needs to be recaptured. The black dots in Figure 7 represent the various joints of the human hand fingers. Once the Leap Motion successfully detects the correct hand information, pressing the “Shift” key displays the image captured by Leap Motion; pressing the “Enter” key initiates automatic data collection; and pressing the “Space” key terminates the program and exports the data to a .txt document.

**Figure 8:**
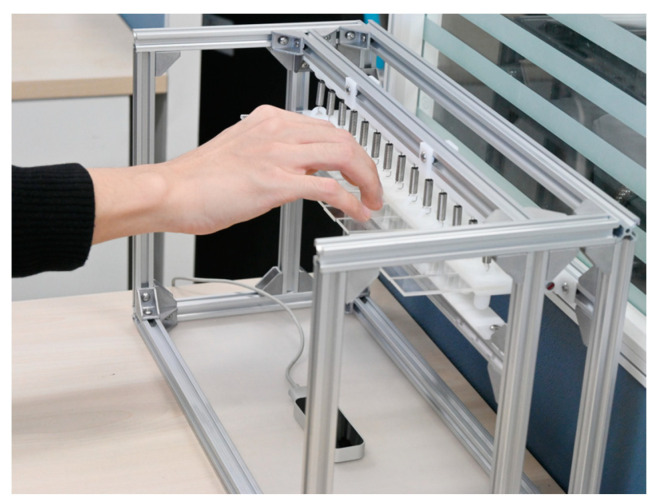
Platform for motion measurement.

To minimize the impact of external obstructions on the experiment and provide participants with a near-realistic piano-playing environment, a motion measurement platform was constructed. As shown in Figure 8, the multi-finger coordinated keystroke motion measurement platform includes a glass surface, simulated piano keys, the Leap Motion sensor, etc. The platform’s frame provides the appropriate height for hand motion measurement, with the Leap Motion positioned directly beneath the fingertip of the middle finger, ensuring the hand remains within the optimal detection range of the Leap Motion sensor [41]. The simulated piano keys, fitted with springs at the joints, have a width of 23.5 mm and a depth of 9.5 mm, corresponding to the standard width and depth of white keys on a piano. During measurement, participants position their fingers on the respective key locations to simulate the standard hand positioning used in piano key-pressing. Subsequently, the ring and middle fingers repeatedly perform coordinated key-pressing actions. Concurrently, the Leap Motion sensor collects positional data of the finger feature points (joints and fingertips of the middle and ring fingers).

### 3.3. Analysis of the Model for Single Individual

This study selected a participant (with a ring finger length of 80.3 mm and a middle finger length of 84.8 mm) as the subject. During the experiment, the participant was first required to position their fingers on the corresponding keys, ensuring that the distal joints of the four fingers, excluding the thumb, were perpendicular to the keys. Following the rhythm set by a metronome (approximately completing one action cycle every 0.75 s), the participant executed 25 consecutive multi-finger coordinated keystrokes actions using the middle and ring fingers. Every 25 consecutive keystroke actions constituted one set of data, and after completing one set of data, participants took a three-minute break. The Leap Motion visual sensor repeatedly captured the motion data of the ring and middle fingers during 25 consecutive keystrokes by the participant. In the measurement experiment, 100 sets of keystroke data from this volunteer were collected, totaling 2500 complete keystroke actions.

**Figure 9:**
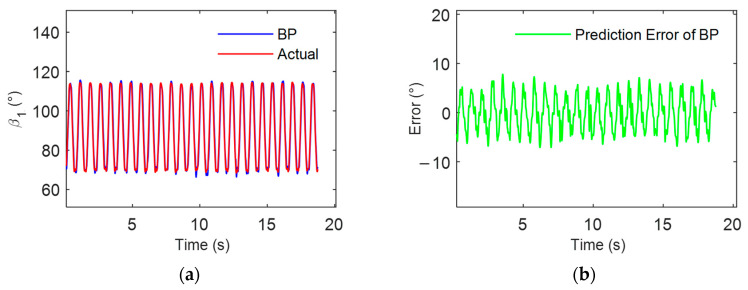
Analysis of individual model for multi-finger coordinated keystroke: (**a**) BP model prediction results and actual values; (**b**) BP model prediction error.

To analyze the coordination of multi-finger coordinated keystrokes in an individual, initially, a model for the participant’s keystrokes was established based on the BPNN. The dataset consists of 100 sets of keypress action data collected during the measurement experiment. The first 80 sets of data were selected for training the BPNN model, while the last 20 sets were used as a validation set. The model’s input parameters included the middle finger MCP joint’s position rotation angle, angular velocity, and angular acceleration, while the output parameter was the ring finger MCP joint’s rotation angle ($\beta_{1}$). The neural network is configured with tansig activation for the hidden layers, purelin activation for the output layer, a learning rate of 0.01, 1000 training epochs, mean squared error (MSE) as the error function, and a batch size of 32. Subsequently, the established model was used to predict actual keystroke actions. The ring finger MCP’s measured motion curve, which refers to the angle curve of the MCP joint of the ring finger as it changes over time during 25 consecutive keystroke actions, was compared with the model’s predicted results. The results are illustrated in Figure 9.

The model prediction errors in Figure 9 demonstrate good consistency between the predicted results of the multi-finger coordinated keystroke model and the actual rotation angle of the ring finger MCP clearly. The root mean square error (RMSE) was only 4.8925°. To further validate the effectiveness of the multi-finger coordinated keystroke model for individuals, the study conducted keystroke action measurements and developed models for the remaining 11 participants. It was found that the RMSE of the model predictions for all participants was less than 5°. These results indicate that in the multi-finger coordinated keystroke actions of an individual, there exists a model for the movements of the ring and middle fingers. This model allows for the inference of the ring finger MCP joint’s rotation angle and angular velocity from the middle finger’s keystroke motion information, enabling the prediction of the ring finger MCP joint’s keystroke motion information.

### 3.4. Analysis of the Model for Multiple Individuals

Building upon the model of multi-finger coordinated keystroke actions for a single individual, this study endeavors to explore the uniformity across different individuals and establish a universal model applicable to multiple individuals. When considering multiple participants, it is necessary to evaluate the potential impact of individual differences on the model. This paper integrates data reflecting individual differences into the multi-individual model. The objective is to alleviate the effects of factors such as individual finger length and piano-playing experience. In the input data of the multi-individual model, besides the rotation angle, angular velocity, and angular acceleration of the middle finger’s MCP joint, additional information, such as the lengths of the middle and ring fingers and years of piano training, is also incorporated. The output of the model remains the rotational angle ($\beta_{1}$) of the ring finger’s MCP joint. This facilitates the establishment of a cross-individual, feature-spanning, multi-individual, multi-finger model.

**Figure 10:**
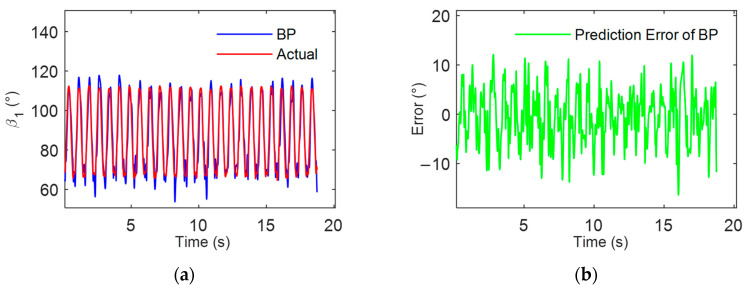
Analysis of multi-individual model for multi-finger coordinated keystroke: (**a**) BP model prediction results and actual values; (**b**) BP model prediction error.

The data samples were obtained from motion tests of 12 participants, labeled from data01 to data12. Each participant contributed 30 sets of keystroke data, with each set comprising motion information from 25 consecutive keystrokes. This resulted in a total of 9000 complete keystroke actions. Additionally, the data samples included individual characteristics such as the lengths of the middle and ring fingers and years of piano training. Model training was conducted using the sample data from data01 to data11, and the constructed model was tested and evaluated using the sample data from data12. The predictive results of the multi-individual universal model are illustrated in Figure 10.

The experimental results indicated that the multi-individual, multi-finger coordination neural network model, constructed based on a BPNN, demonstrated good consistency with the actual measured rotational angles, with an RMSE of 7.9053°. This outcome further confirms that a unified motion model for the coordinated keystroke action of the middle and ring fingers exists among individuals following prolonged professional piano key training. The experimental sample comprised 12 individuals who had undergone several years of professional piano training, possessing similar characteristics such as duration of piano training, which might be the reason for the observed regularity in their multi-finger coordinated keystroke actions.

**Figure 11:**
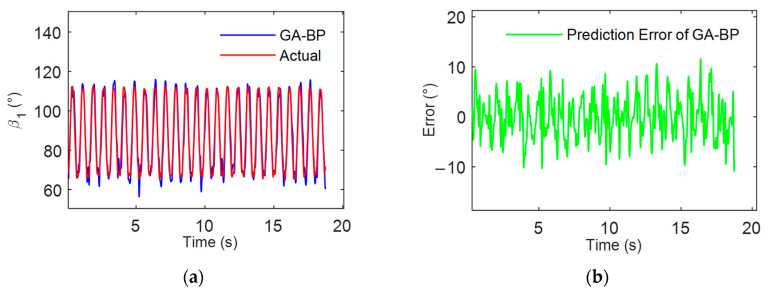
Analysis of multi-individual model for multi-finger coordinated keystroke based on GA-BP: (**a**) GA-BP model prediction results and actual values; (**b**) GA-BP model prediction error.

**Figure 12:**
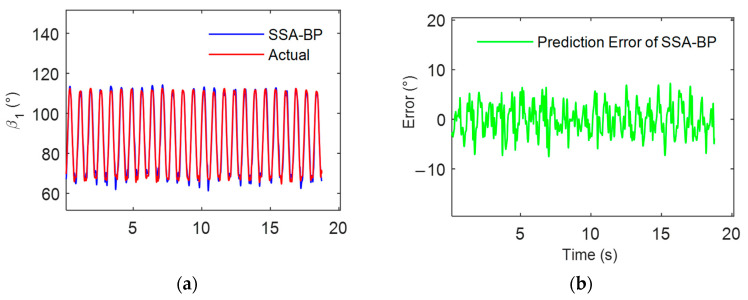
Analysis of multi-individual model for multi-finger coordinated keystroke based on SSA-BP: (**a**) SSA-BP model prediction results and actual values; (**b**) SSA-BP model prediction error.

This study attempts to further reduce the error in the multi-individual multi-finger coordinated keystroke action model. The model is optimized using both a genetic algorithm (GA) and sparrow search algorithm (SSA), with the process illustrated in Figure 8. The data samples were similarly selected from motion tests labeled data01 to data12. Model training was conducted using sample data from data01 to data11, and the model was tested and evaluated with sample data from data12. The predictive results of the multi-individual multi-finger model based on GA-BP and SSA-BP are, respectively, shown in Figure 11 and Figure 12.

The experimental results demonstrated that the BPNN model, optimized with the improved algorithm, exhibited higher consistency between its predictions on the data12 sample and the actual measured data. Compared to the model constructed using a single BPNN, those based on GA-BP and SSA-BP showed improved prediction accuracy. The RMSE was reduced to 6.1729° and 4.8328°, respectively, indicating that the improved algorithms enhance the accuracy of the multi-individual model. Furthermore, as evidenced by Figure 10, Figure 11 and Figure 12, the SSA-BP model exhibited smaller fluctuations in prediction error compared to the BP and GA-BP models.

**Figure 13:**
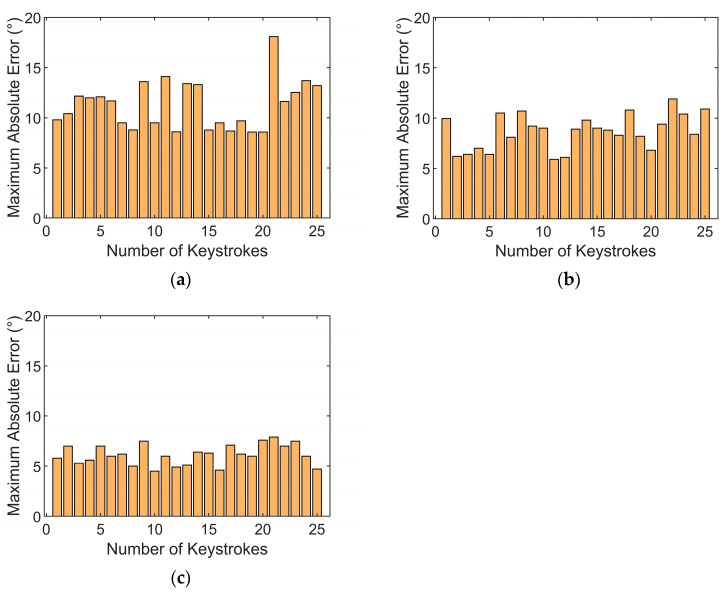
Histogram of maximum absolute errors for each cycle of each coordinated model: (**a**) BP model; (**b**) GA-BP model; (**c**) SSA-BP model.

The histograms of the maximum absolute errors across various keystroke motion cycles as depicted in Figure 13. It is observed from the histograms that the BP model demonstrates greater dispersion and higher peaks in the maximum absolute errors across various keystroke motion cycles, indicating potential large errors in certain predictive scenarios. This reflects the limitations of traditional BPNNs regarding parameter initialization and susceptibility to local optima. When compared with the BP model, the GA-BP model displays a distribution of maximum absolute errors with lower peaks and a trend toward reduced variability, albeit with some fluctuations. This indicates that the genetic algorithm enhances the overall predictive accuracy and stability of the BP neural network to a certain extent. The SSA-BP model exhibits the most uniform and lowest peaks in the distribution of maximum absolute errors, demonstrating superior performance even in the worst-case prediction scenarios. These findings underscore the effectiveness of the sparrow search algorithm in improving the predictive accuracy of the BP neural network and in mitigating extreme errors.

## 4. Discussion

Understanding the coordination of human motion has long been a persistent issue. The middle and ring fingers of humans are subject to significant neural and biomechanical constraints. In this study, the existence of a coordinated multi-finger keystroke action model, particularly for professional pianists’ middle and ring fingers, was identified. The degrees of freedom of normal finger joints were first analyzed, and the flexion movement of fingers in multi-finger piano keystroke actions was simplified. Two-dimensional angular parameters ($\theta_{1}$ and $\beta_{1}$) were used to characterize the rotational angle information of the MCP joints of the middle and ring fingers. Subsequently, a keystroke action measurement environment was established, involving the secondary development of the Leap Motion sensor and the design of a measurement interaction interface. A BP neural network-based multi-finger coordinated keystroke action model suitable for individual subjects was then developed. Finally, based on the individual model, individual characteristic information was further considered. The SSA and GA were introduced in the model development process, leading to the establishment of multi-individual, general multi-finger coordinated keystroke action models based on BP, GA-BP, and SSA-BP neural networks.

In the structural design of BPNNs, the number of hidden layers is a key factor. While increasing the number of hidden layers can enhance the accuracy of BPNNs, it also leads to increased complexity in network training and prolonged training durations [42]. Previous studies have commonly suggested that a single hidden layer in BPNNs is capable of approximating various nonlinear continuous functions with high precision [43]. Consequently, the BPNN used in this study was configured with a single hidden layer. The number of neurons in the hidden layer is equally crucial for model performance. Too few neurons may prevent the BPNN model from converging to the desired accuracy, while too many may lead to overfitting of the training data [44]. Therefore, based on prior research [44,45], this study determined, via trial and error, that the optimal number of neurons in the hidden layer is 13. In the BPNN-based individual multi-finger coordinated keystroke action model, the model’s predictive results showed good consistency with the actual rotational angles of the ring finger’s MCP, with root mean square errors less than 5°, indicating high overall predictive accuracy. The likely reason is that the coordinated multi-finger keystroke actions of an individual are stimulated and coordinated by central pattern generators [46], exhibiting strong regularity. This enables BPNN to effectively simulate the human brain in processing fuzzy mapping relationships and recognizing complex nonlinear relationships between variables [47].

To comprehensively assess the predictive accuracy of the multi-individual model, this study selected mean absolute error (MAE), root mean square error (RMSE), and mean absolute percentage error (MAPE) as evaluation metrics for the model. The analysis of data in Table 3 revealed that, compared to the individual model, the RMSE of the BPNN-based multi-individual model increased by approximately 58%. The likely reason for this increase is the higher complexity of multi-finger coordinated keystroke action data across multiple individuals, leading to an expanded dimensionality of the feature space. In high-dimensional spaces, finding the global optimum becomes more challenging [48], potentially causing the BPNN model to converge to local optima. Furthermore, the assessment results of different models indicate that the optimized BP model, enhanced by optimization algorithms, outperforms the standalone BP model in terms of predictive effectiveness. This outcome validates the effectiveness of the GA and SSA in optimizing the weights and thresholds of the BP neural network, effectively preventing the model from becoming trapped in local optima, thereby enhancing the model’s predictive accuracy and stability. Specifically, the MAE, RMSE, and MAPE of the SSA-BP model were 3.8127°, 4.8328°, and 4.4371%, respectively, with its prediction errors and variability being lower than those of the GA-BP and BP models. This demonstrates SSA’s stronger robustness in finding global optima, consistent with its application outcomes in other fields [49].

In summary, among the three multi-individual coordinated keystroke action models, the SSA-BP model demonstrates the most stable prediction results and the highest predictive accuracy. It exhibits strong predictive capabilities, accurately forecasting the keystroke actions of professional pianists. Additionally, the keystroke variation curves of professional pianists can serve as standard keystroke curves. For instance, in the assessment of the effectiveness of multi-finger coordination teaching in piano instruction, by measuring individual characteristic information and middle finger MCP angle data of piano beginners, it is possible to predict the expected variation curve of the ring finger’s MCP for corresponding professional pianists. By comparing the model’s predicted curve with the actual keystroke action curve of the ring finger’s MCP of piano beginners, scientific and intuitive motion guidance and error correction can be provided for the training of multi-finger coordinated keystroke action coordination for piano beginners. Beginners can then train to bring their own keystroke action curves closer to the model’s predicted curve. Furthermore, this research can be applied in areas such as robotic hand motion planning for piano instruction and intelligent assistive devices, actively promoting the development of these fields.

This study still has several limitations. Firstly, the subjects in the measurement experiment were all young people. Future studies would collect data from professional pianists of various age groups to enhance the model’s efficacy. Secondly, the sample size of this study was small, involving data collection from only 12 professional pianists for multi-finger coordinated keystroke actions, unsuitable for training with complex neural networks. Consequently, subsequent studies could expand the model’s application scope by increasing the total number of participants and integrating other characteristic information of various age groups, such as individual age, height, and profession, to construct a keystroke action model suitable for diverse populations. This multi-individual, multi-finger coordinated keystroke action model could be established as a more broadly applicable cross-individual, cross-characteristic general model by incorporating additional individual information beyond finger length and years of piano training. Finally, the current study focused solely on coordinated keystroke actions with a 0-degree phase difference. Future research could explore coordinated keystroke actions with other phase differences.

## 5. Conclusions

This paper focuses on the coordinated finger keystroke actions of the middle and ring fingers, proposing a modeling method for a multi-finger coordinated keystroke action model based on the backpropagation neural network optimized by the sparrow search algorithm (SSA). The effectiveness of this model is demonstrated by experimental results. Comparative analysis of prediction errors indicates that the SSA-BP model surpasses the BP and GA-BP models in predictive accuracy, with a root mean square error of less than 5°. Using this model, the motion information of the ring finger’s metacarpophalangeal (MCP) joint can be accurately predicted based on the motion information of the middle finger. Additionally, this research not only provides training guidance and evaluation methods for beginners in multi-finger keystroke actions but also offers methodological references for more complex piano-playing actions, such as cross-fingering and finger ensemble coordination.

## Figures and Tables

**Table 1:** Parameters setting of GA.

Population Size	Number of Generations	Crossover Probability	Mutation Probability
50	100	0.6	0.2

**Table 2:** Parameters setting of SSA.

Population Size	Number of Iterations	Explorer Ratio	Vigilante Ratio
50	100	0.7	0.2

**Table 3:** Evaluation metrics data for multi-individual models.

Model	MAE (°)	RMSE (°)	MAPE/%
BP	6.2713	7.9053	7.1704
GA-BP	4.7539	6.1729	6.4912
SSA-BP	3.8137	4.8328	4.4371

## Data Availability

The data cannot be made public due to ethical reasons and will be provided upon reasonable request.
